# Revealing Progressive Degradation of Cobalt Oxide Nanoparticles During Thermochemical Redox Cycling via Operando STEM-EELS

**DOI:** 10.1021/acs.nanolett.5c05081

**Published:** 2025-12-17

**Authors:** Madeline Van Winkle, Stephen D. House, Yuxiang Peng, Yu-chen Karen Chen-Wiegart, Katherine Jungjohann, John S. Mangum

**Affiliations:** † Materials Science Center, 53405 National Laboratory of the Rockies (formerly National Renewable Energy Laboratory), Golden, Colorado 80401, United States; ‡ Center for Integrated Nanotechnologies, 1105 Sandia National Laboratories, Albuquerque, New Mexico 87123, United States; ¶ Department of Materials Science and Chemical Engineering, 12301 Stony Brook University, Stony Brook, New York 11790, United States; § National Synchrotron Light Source II (NSLS-II), Brookhaven National Laboratory, Upton, New York 11973, United States

**Keywords:** thermochemical material, energy storage, operando electron microscopy, metal oxide, EELS

## Abstract

Metal oxides are promising materials for long-duration thermochemical energy storage. Efforts to characterize their reaction kinetics, conversion rate, and morphological evolution during thermochemical cycling have largely focused on bulk and microscale measurements. However, the design of nanostructured metal oxides could improve the reaction reversibility and kinetics, warranting the development of platforms to investigate how these materials behave at the nanoscale. Here, we demonstrate the use of correlative, time-resolved electron energy loss spectroscopy and imaging in an environmental transmission electron microscope for studying the thermochemical cyclability of cobalt oxide nanoparticles with high spatial and temporal resolution. The spectroscopic data reveal a striking decrease in reaction kinetics after the first cycle, resulting from sintering-driven nanostructural densification. Comparison between cycling in humid and dry air shows that atmospheric conditions can modulate reaction transition temperatures but have limited effects on sintering over multiple cycles, suggesting long-term durability will instead rely on synthetic and/or nanostructural modifications.

## Graphical abstract

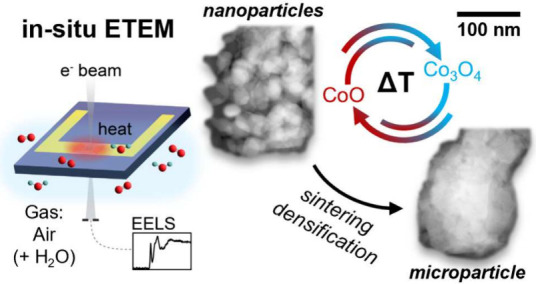

The development of materials for thermal energy storage (TES) is crucial for optimizing energy capture and usage in cooperation with various energy production technologies. These TES materials are key to industrial applications^1^ for efficient energy management, waste heat recovery, and cost reduction and central to both solar^2^ and next-generation nuclear power plants^3^ for providing stable and flexible power. Thermochemical materials (TCMs), which store and release thermal energy through reversible chemical reactions, have emerged as a particularly promising class of materials for TES due to their relatively high energy storage densities and long storage durations.^4^ In this work, we focus on cobalt oxide, a model TCM system with an energy storage density of 844 kJ/kg,^5^ which undergoes a thermally induced reduction–oxidation (redox) process where the reduction to CoO is endothermic and oxidation to Co_3_O_4_ is exothermic, as described in eq 1 and Figure 1a.

1 $$2{\mathrm{Co}}_{3}O_{4}\left(s\right)+\Delta H↔6\mathrm{CoO}\left(s\right)+O_{2}\left(g\right)$$

Under constant pressure, increasing temperature drives the reduction to CoO, releasing oxygen from the lattice as O_2_ gas. Conversely, decreasing temperature leads to reincorporation of oxygen to form Co_3_O_4_.^6^ Since the gaseous species in this reaction is O_2_, cobalt oxide (and other metal oxide redox pairs) can be thermochemically cycled in ambient air. This provides a considerable practical advantage compared to other TCM systems, which involve different gaseous reactants/products (e.g., H_2_O, CO_2_, etc.) that may require energetically costly phase changes, separation, and/or containment during operation.^7–10^

**Figure 1:**
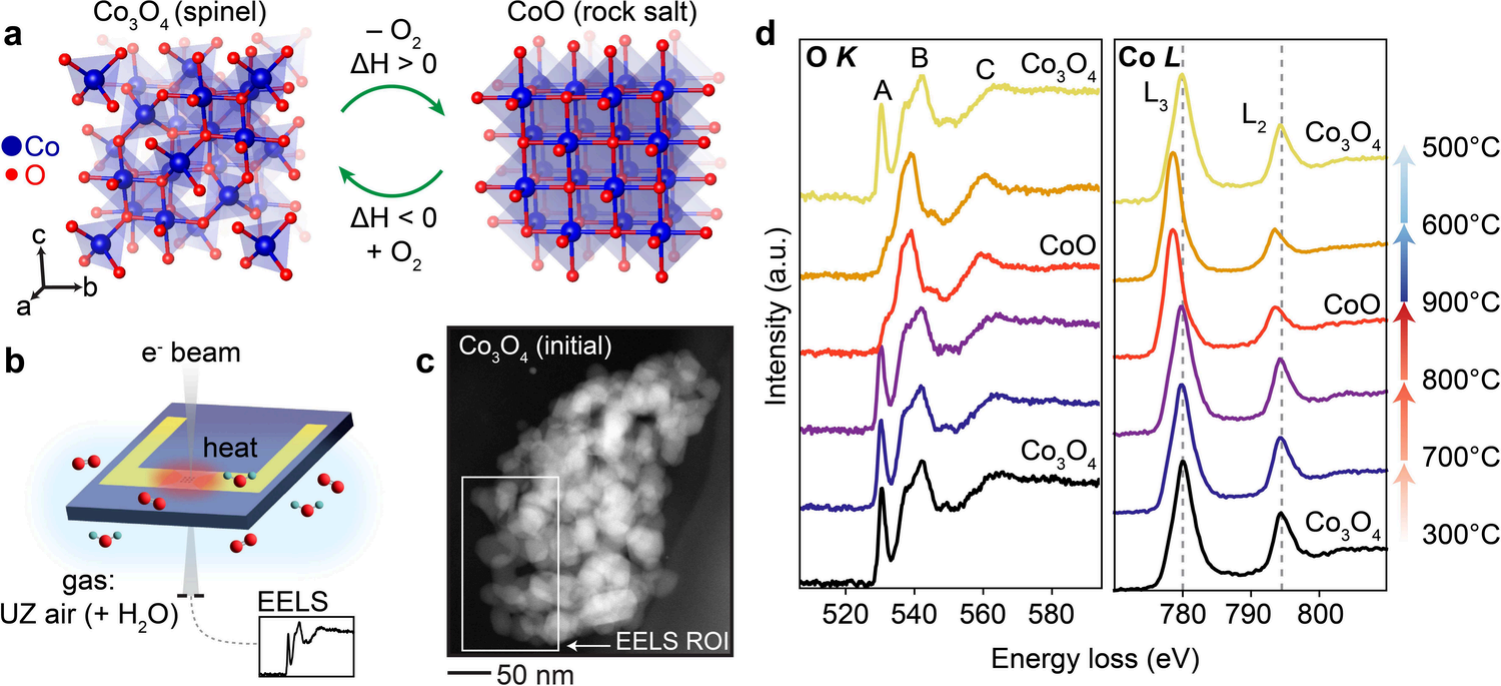
(a) Crystal structures for Co_3_O_4_ and CoO. Conversion between phases is temperature-driven. (b) Schematic of the experimental setup in the ETEM. (c) ADF-STEM image of the Co_3_O_4_ nanoparticle cluster prior to thermochemical cycling. (d) Summed EEL spectra (from spectral images collected in the region outlined in (c)) at various temperatures during the first cycle. The sample equilibrated at each temperature for 20–30 min prior to data collection. Changes in the O K-edge and Co L_3,2_-edge indicate reduction to CoO upon heating from 300 to 900 °C and reoxidation to Co_3_O_4_ upon cooling from 900 to 500 °C under 0.5 mbar Ultra Zero (UZ) grade air with added 38% relative humidity.

Thus far, most studies on cobalt oxide-based TCMs have focused on characterizing bulk thermal properties using thermogravimetric analysis and differential scanning calorimetry,^7,9,11,12^ as well as microscale structure and composition using scanning electron microscopy^7,9,11,12^ and X-ray diffraction.^9,11,12^ It has been proposed that nanostructured thermochemical materials could offer better reaction reversibility and faster conversion kinetics than microparticles owing to their high surface area to volume ratios,^7^ as has proven beneficial in other energy storage technologies.^13,14^ However, relatively little high-resolution characterization has been performed on TCMs in general, and especially for thermochemical nanomaterials with particle sizes below 100 nm.^15^ For cobalt oxide, several studies have used (scanning) transmission electron microscopy [(S)­TEM] to demonstrate either the reduction of Co_3_O_4_ to CoO and/or Co^16–21^ or the complementary oxidation process^22,23^ in nanoparticles, but have not explored reaction reversibility or changes over repeated thermochemical cycling, a fundamental understanding of which is critical for TES applications.

Here, using environmental TEM (ETEM) we investigate the nanostructural and chemical evolution of cobalt oxide nanoparticles during multiple thermal redox cycles. We demonstrate how operando electron energy loss spectroscopy (EELS) can be used to quantify the conversion between redox states in nanoscale metal oxides with high spatial and temporal resolution during thermal cycling, revealing a notable decrease in the reaction kinetics after the first cycle. These changes are directly correlated to marked sintering and nanostructural densification observed with concurrent annular dark-field scanning transmission electron microscopy (ADF-STEM) imaging. In addition, we identify the effects of water vapor in the surrounding atmosphere on the reaction transition temperatures and particle sintering, revealing a change in oxidation temperature in humid versus dry conditions but limited long-term impacts on nanostructural degradation. These results highlight major challenges that must be addressed in order to harness the potential benefits of metal oxide nanoparticles for TES and offer a perspective on the material design factors that are likely to be most influential in realizing this goal.

Figure 1b shows a schematic of the experimental setup. Samples were prepared by drop casting Co_3_O_4_ nanoparticles dispersed in isopropanol onto Protochips Fusion Select heating chips with a 40 nm thick silicon nitride membrane (further details included in Supporting Information). Transformations in the nanoparticles were then induced by flowing an air–water vapor mixture (0.5 mbar) with 38% relative humidity through the ETEM while varying the temperature. While laboratory-scale thermochemical cycling experiments for metal oxide TCMs are typically run under ambient air, which contains some moisture, the standard Ultra Zero (UZ) grade air used for ETEM experiments has extremely low moisture levels (0% relative humidity). In this experiment, water vapor was intentionally and controllably mixed with UZ air introduced into the microscope to more closely replicate realistic operating conditions used for other experiments in laboratory settings or for commercial application. We also note that although the individual Co_3_O_4_ nanoparticles are around 20–50 nm in diameter, they aggregated into clusters upon drop casting (Figure 1c), which better represents the environment inside an actual thermochemical reaction chamber. The thermochemical cycling behavior shown in this work therefore results from both intrinsic factors and multiparticle interactions, which will be discussed in further detail.

To identify the reduction and oxidation temperatures in the chosen atmospheric conditions, the temperature was changed incrementally, holding for 20–30 min at each target temperature prior to EELS acquisition. Reversible conversion between Co_3_O_4_ and CoO states is evident in the EEL spectra shown in Figure 1d, which correspond well to literature references.^16,21^ These spectra represent the sum of all pixels from the spectral images collected at each temperature point in the cluster region outlined in Figure 1c. Upon heating, reduction from Co_3_O_4_ to CoO is observed around 900 °C. Looking first at the Co features, there is a shift in both the Co L_3_- and L_2_-edges from 780.1 and 794.5 eV, respectively, in Co_3_O_4_ to 778.6 and 793.7 eV in CoO. There is also a decrease in intensity of the L_2_-edge relative to that of the L_3_-edge upon conversion to CoO. Both changes correspond to the shift from a mixed Co­(II/III) valence state in the Co_3_O_4_ spinel structure to Co­(II) in the CoO rock salt. At the O K-edge, there is a strong pre-edge peak (labeled A) at 530.7 eV in the Co_3_O_4_ spectra that weakens considerably after reduction to CoO. This peak is attributed to transitions into hybridized O 2p–Co 3d states and is larger for Co_3_O_4_ due to increased covalent hybridization of the Co­(III) sites present.^21,24^

Once in the CoO state, oxidation back to Co_3_O_4_ requires a substantial decrease in temperature to around 500 °C, indicating a relatively large thermal hysteresis of 400 °C. Here, thermal hysteresis refers to the difference between the reduction and oxidation temperatures. Such hysteresis has been reported previously in metal oxides^7,25^ and typically has a greater impact on the oxidation process compared to reduction, requiring more extensive cooling below the equilibrium temperature to provide enough thermal driving force for reoxidation to occur.^26^ Previously reported bulk thermal measurements on cobalt oxide powders have demonstrated thermal hysteresis with a magnitude below 50 °C.^7,27,28^ We attribute the much lower reoxidation temperature and larger hysteresis in our work primarily to the lower operation pressure (and therefore lower O_2_ partial pressure), which increases favorability of the reduced state^29^ and has been shown to widen thermal hysteresis in other metal oxides.^26^ We also acknowledge that exposure to relatively high electron beam doses, as is often the case in EELS experiments, can have a reducing effect^21^ that could also inhibit reoxidation. However, lower magnification images of the cluster acquired periodically during cycling (Supplementary Figure 1) show consistent morphological changes between the region sampled with EELS and surrounding areas with minimal beam exposure (only exposed for a single-pass ADF-STEM image after each temperature equilibration period). This suggests that, on the length scale studied in this work, the transition temperatures observed and accompanying nanostructural changes that occur, discussed later, are not noticeably impacted by the levels of beam exposure used here. Still, electron beam dose is an important factor to consider when using the methodology employed in this work on more beam sensitive systems.

With the reduction and oxidation transition temperatures identified, we investigated the kinetics of each conversion process in more detail using time-resolved EELS. To capture these processes, the temperature was either raised or lowered at a ramp rate of 1 °C/s near the transition (from 800–900 °C for the reduction and from 600–500 °C for the reoxidation) and then held at the target transition temperatures (900 and 500 °C) for around 10 min. During both the temperature ramp and the subsequent hold, EEL spectral images of the cluster (from the region outlined in Figure 1c) were collected with a fast per-pixel exposure time (1 ms), yielding a total temporal resolution of 2.4 s/spectral image. The plots shown in Figure 2a,b illustrate the evolution of both the O and Co spectral features over time during Cycle 1, where each plotted spectrum is the sum of all pixels in five consecutive spectral images (resulting in 12 s of temporal resolution). During the reduction, the aforementioned changes in the O K-edge prepeak (A) and Co-L_3,2_ edge become clear starting around 1 min after reaching 900 °C. Meanwhile the subsequent reoxidation is slower, which aligns with what has been observed by thermogravimetric analysis.^11^ Here, obvious signs of reoxidation emerge around 6 min after reaching 500 °C.

**Figure 2:**
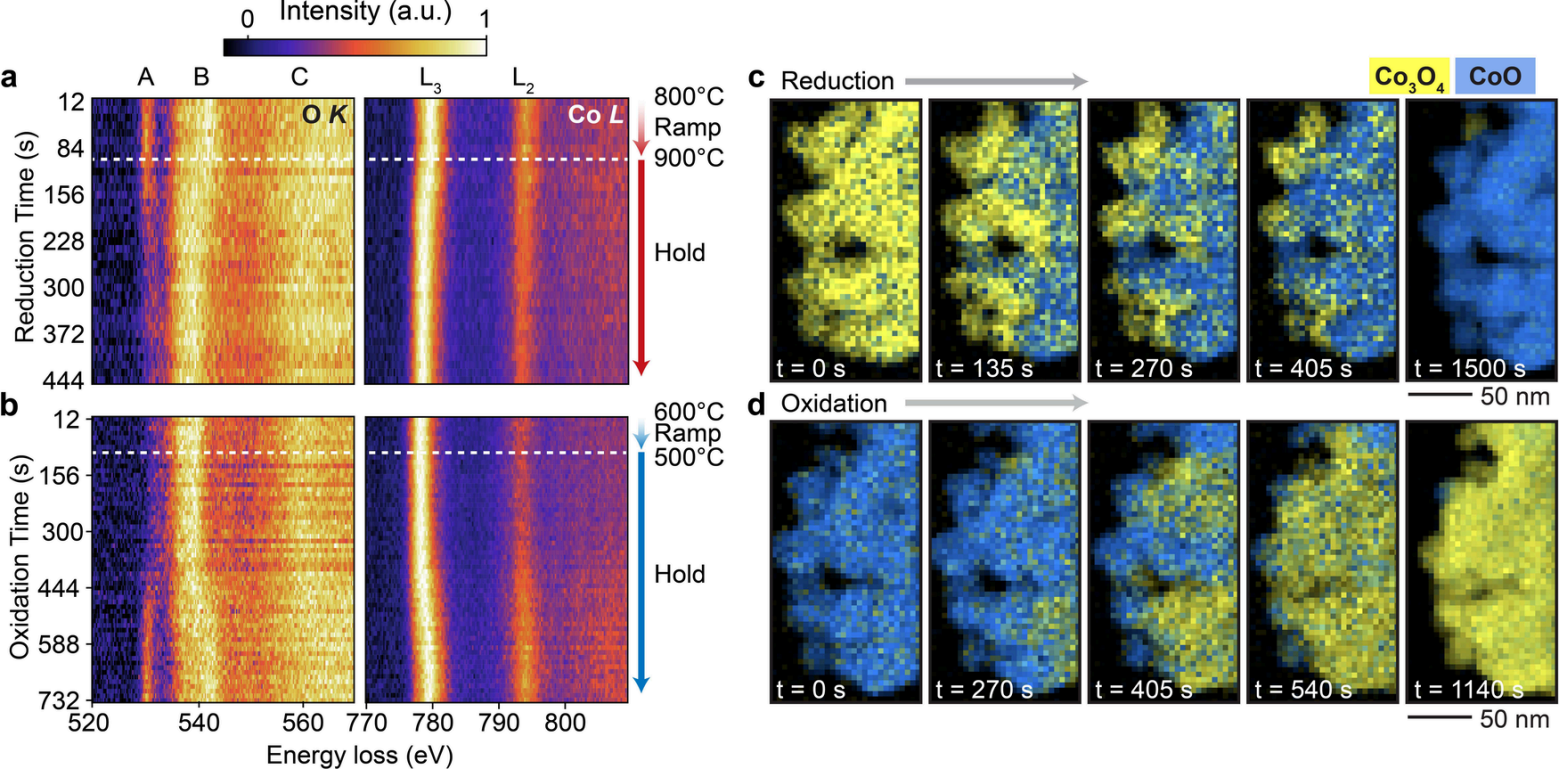
Time-resolved EELS showing evolution of the O K-edge and Co L_3,2_-edge during the first (a) reduction at 900 °C and (b) reoxidation at 500 °C. Plotted spectra are the sum of all pixels in five fast-exposure EEL spectral images (2.4 s/spectral image, yielding 12 s time steps). (c,d) Corresponding phase maps of the nanoparticle cluster at various time points, illustrating nucleation and growth of the CoO phase (blue) during reduction (c) and the Co_3_O_4_ phase (yellow) during reoxidation (d). Phases were distinguished by MLLS fitting using internal references and the individual phase maps were overlaid such that the color represents the majority phase at each pixel.

While the single-pixel signal-to-noise ratio in the individual fast-acquisition spectral images is too low for quantification, summing a larger number of frames over time (56 frames in this case) overcomes this challenge and enables clear spatial visualization of the reaction progress. Figure 2c,d contains maps showing the change in distribution of Co_3_O_4_ and CoO phases in the nanoparticle cluster during the reduction (Figure 2c) and reoxidation (Figure 2d) processes. In these maps, the majority phase at each pixel was determined using a multiple linear least-squares (MLLS) fitting with internal Co_3_O_4_ and CoO references (see Supporting Information). In both cases, conversion nucleates at similar points in the cluster and gradually spreads outward until the majority of the cluster is converted, after around 20–25 min. The underlying cause of these preferred nucleation sites warrants further investigation in future studies; however, one possible cause is a statistical effect, where certain areas of the cluster have a higher density of nanoparticles and reactive surfaces present and therefore a higher probability of reacting earlier (see Supporting Information for further discussion). While we have chosen to focus here on the thermochemical cycling behavior of nanoparticle clusters to more closely resemble the macroscale state found in a thermochemical reaction chamber, future fundamental investigation of nucleation behavior in dispersed single particles could provide an insightful comparison to test this hypothesis. Additionally, more factors that are unfeasible to investigate within the scope of this work could also influence where the thermochemical reaction begins (e.g., the cluster’s thermal contact area with the MEMS heater chip and fluid dynamics of the reactive gas around the cluster).

A key factor for the practical use of TCM systems is their durability over multiple thermochemical cycles. With this in mind, we compared the reduction and oxidation kinetics for the first two thermal cycles. Figure 3 shows the change in the CoO fraction in the cluster over time for the first two cycles, where the CoO fraction was again calculated using MLLS fitting. These results show a stark change in behavior after even a single thermochemical cycle. In the first reduction process, two kinetic regimes, one faster (100–200 s) and one slower (after 200 s), are observed, suggesting a crossover from a reaction-limited to a diffusion-limited time period, respectively. Two regimes are also observed during the second reduction (between 100–400 s and after 400 s); however, there is a decrease in the slope of the first regime, suggesting heat transfer through the bulk of the material may be prolonged in the second cycle.^15^ Despite this, a similar CoO fraction is reached in both reduction cycles, with around 80–85% conversion to CoO 6–7 min after reaching 900 °C and near 100% conversion after longer time intervals (15–20 min).

**Figure 3:**
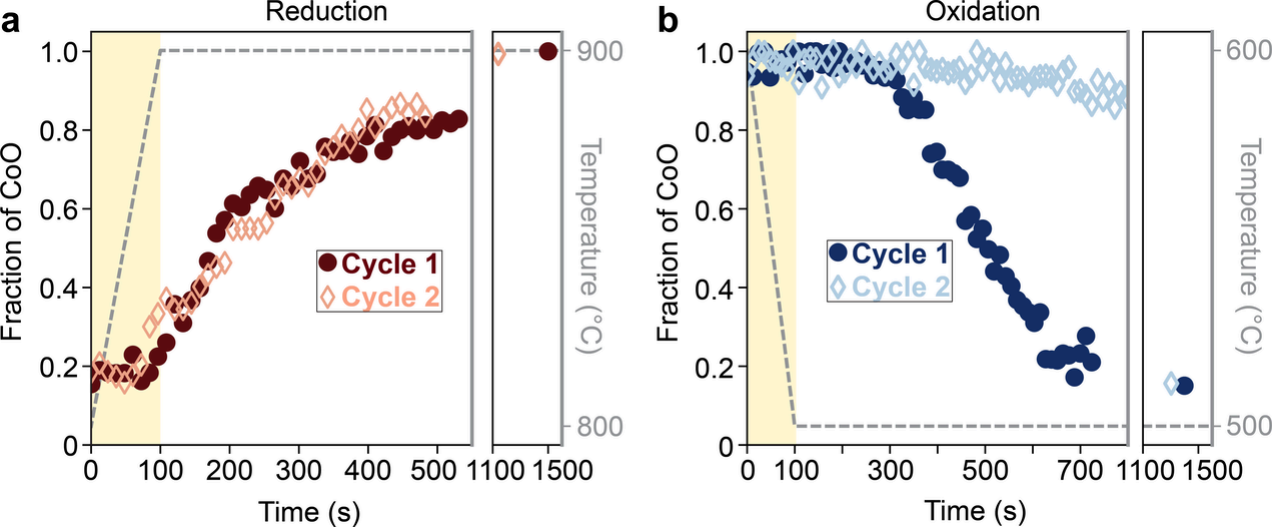
Comparison of conversion kinetics during the first two thermal cycles. Plots show change in fraction of CoO in the nanoparticle cluster over time during the first and second (a) reduction and (b) reoxidation, highlighting a degradation in kinetics in the second cycle, particularly during reoxidation. Corresponding temperature profiles are shown in gray.

On the other hand, the reoxidation process (Figure 3b) shows not only a change but also a considerable degradation in kinetics in the second cycle. While the remaining CoO fraction begins to stabilize around 15–20% 8 min after reaching 500 °C during the first reoxidation, the remaining CoO fraction is still about 95% at this time in the second cycle. After 20 min, the majority of the cluster has ultimately reoxidized in the second cycle, but the substantially more sluggish kinetics compared to the first cycle stands out as problematic nonetheless. This discrepancy between the two cycles likely results from the greater distance over which diffusion must occur for Co ions in the solid^22,23^ and less active area for O_2_ from the surrounding atmosphere to react during the second cycle, due to factors including particle coarsening and decreasing surface area-to-volume ratio. The reliance of the reaction progress on oxygen uptake is also evident from the change in the elemental composition of the cluster during cycling (Supplementary Figure 5). Although the presence of significant background oxygen signal from the surrounding gas impedes pure quantitative analysis of atomic composition from the EELS data, there is a relative decrease in oxygen content in the cluster during reduction and an increase during reoxidation. However, in the second cycle, there is minimal change in the oxygen content in the cluster during the first 10 min of reoxidation, consistent with the observation of negligible change in phase fractions from the MLLS fitting.

The degradation in kinetics revealed by the spectroscopic data directly correlates to nanostructural changes in the nanoparticle cluster. Figure 4a,b shows ADF-STEM images of the cluster at various time points during the first reduction (Figure 4a) and reoxidation (Figure 4b). Even in the first cycle, sintering of the nanoparticles and concurrent loss of interparticle porosity is evident. Sintering and nanostructural densification continue to worsen in subsequent cycles until a sintered, single particle, hundreds of nanometers in diameter, has formed by the fifth cycle (Figure 4c).

**Figure 4:**
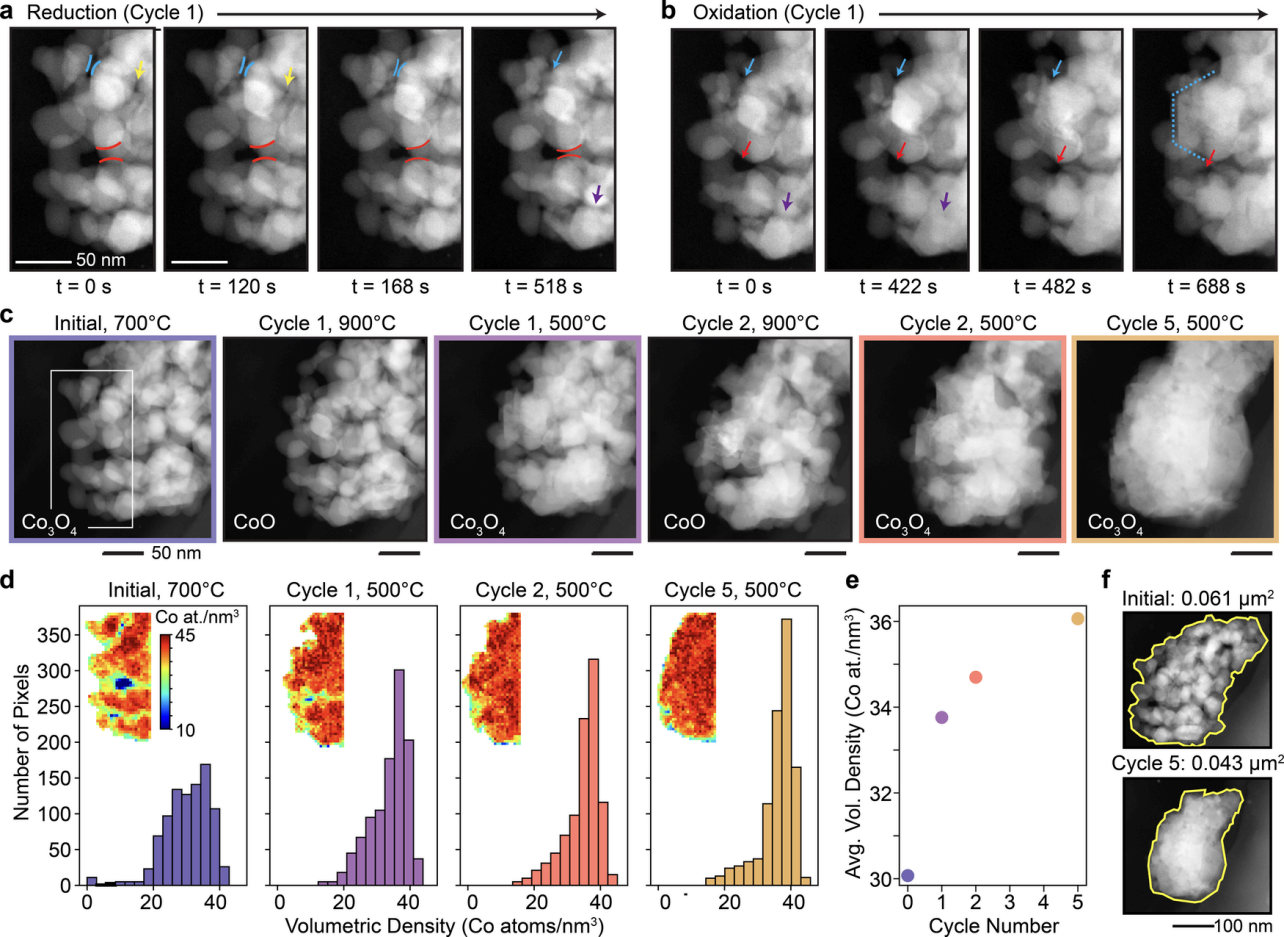
ADF-STEM images showing nanostructural changes in the cobalt oxide nanoparticle cluster during (a) the first reduction, (b) the first reoxidation, and (c) throughout the first five thermochemical cycles. Sintering between particles begins in the first cycle and worsens in subsequent cycles, until a single particle forms. The colored lines and arrows in panels (a) and (b) highlight a handful of points where sintering and/or pore closure are occurring. (d) Histograms, maps, and (e) averages of the volumetric density of Co (Co atoms/nm^3^) during the first five cycles, showing consistent increase in volumetric density due to sintering-driven densification. (f) Initial and final ADF-STEM images of the entire cluster. A ∼30% reduction in the 2D area is observed after five cycles.

Sintering, driven by minimization of the particles’ surface energy,^15,30^ is a commonly reported irreversible phenomenon in microscale thermochemical materials and is known to reduce active surface area and overall cyclability.^27^ Similar findings have also been reported in an ex-situ study on manganese oxide nanoparticles.^15^ The study on manganese oxide noted a size-dependent sintering mechanism where smaller nanoparticles (<100 nm diameter) formed a denser sintered microstructure whereas larger nanoparticles (∼100–300 nm) formed a coarser sintered microstructure with some interparticle porosity maintained. The tendency toward a densified structure in smaller particles can be attributed to their higher surface area-to-volume ratio and more tightly packed initial state. The results in Figure 4 show that cobalt oxide nanoparticles in the size range studied here (<50 nm) are also highly susceptible to sintering-driven densification, suggesting this degradation mechanism is generalizable to many types of metal oxide nanoparticles. Substantial redistribution of mass and closure of open space between particles are reflected in the continual increase in volumetric density of Co atoms with each cycle (Figure 4d,e, see Supporting Information for additional details) as well as the roughly 30% reduction in total area of the cluster after five cycles (Figure 4f), and ultimately lead to the stark changes in conversion kinetics observed after the first cycle.

Furthermore, from watching these transformations *in situ*, it appears that densification occurs to a greater extent during the lower temperature reoxidation process (Figure 4b). This could happen because reoxidation to Co_3_O_4_ leads to volumetric expansion of the nanoparticles, due to a decrease in intrinsic material density and outward diffusion of excess Co that then reacts on the surface.^19,22^ Such expansion would bring grains within the cluster into closer proximity and potentially facilitate sintering. This observation is counterintuitive to the conventional notion that sintering occurs more favorably at high temperatures, typically near the melting point. Limiting or circumventing the effects of volumetric expansion and contraction could, therefore, be critical for maintaining thermochemical cyclability in nanoscale metal oxides. In studies on dense cobalt oxide pellets, lattice expansion and contraction was also noted as problematic, leading to poor structural stability and cracking.^31^ Incorporation of the active material into an inert mesoporous support structure, such as a honeycomb design or porous foam, improved durability.^31–33^ Perhaps comparable open support frameworks (e.g., carbon nanotubes^34,35^ ) could be utilized to improve the nanostructural stability of metal oxides in future work.

Some studies on other TCM systems, such as CaCO_3_/CaO,^36^ have reported that water molecules may facilitate sintering. To investigate whether a water-vapor-free atmosphere would improve the durability of cobalt oxide, we thermochemically cycled a fresh sample of Co_3_O_4_ nanoparticles in 0.5 mbar Ultra Zero (UZ) grade air with 0% relative humidity. The EELS data provided in Figure 5a, which are summed spectra generated from spectral images of the cluster region outlined in Figure 5b, show that the lack of water vapor in the cycling atmosphere did not change the reduction temperature (900 °C) but did raise the reoxidation temperature to around 600 °C, decreasing the magnitude of thermal hysteresis. This may be a result of the increase in surface energy for anhydrous CoO compared to hydrous CoO, increasing the energetic favorability of reoxidation in a dry atmosphere.^37^ Aside from the change in the oxidation temperature, we do not observe notable changes in sintering behavior under dry conditions. While there are some particles at the edge of the cluster that have not fully coalesced by Cycle 5, the cluster is still significantly densified by Cycle 9 (Figure 5b,c). Thus, for the cobalt oxide system, changing the atmospheric composition can influence the driving force and/or activation energy for thermochemical conversion, but for long-term durability, sintering remains a problem to be addressed through adjustments to material composition and/or nanostructure. For example, many studies have explored the use of dopants for mitigating sintering in metal oxide TCMs. While results have varied, incorporation of some species including Al,^9^ Fe,^27^ and Si^38^ has led to improved microstructural stability compared to pure metal oxide counterparts. Similar compositional changes could be beneficial for thermochemical nanomaterials. Sintering in nanoscale metal oxides has also been a concern for catalysis applications. Efforts to address this challenge through design of catalyst support structures with favorable chemical, electronic, and morphological properties^39^ may be transferable to improving TCM cyclability as well.

**Figure 5:**
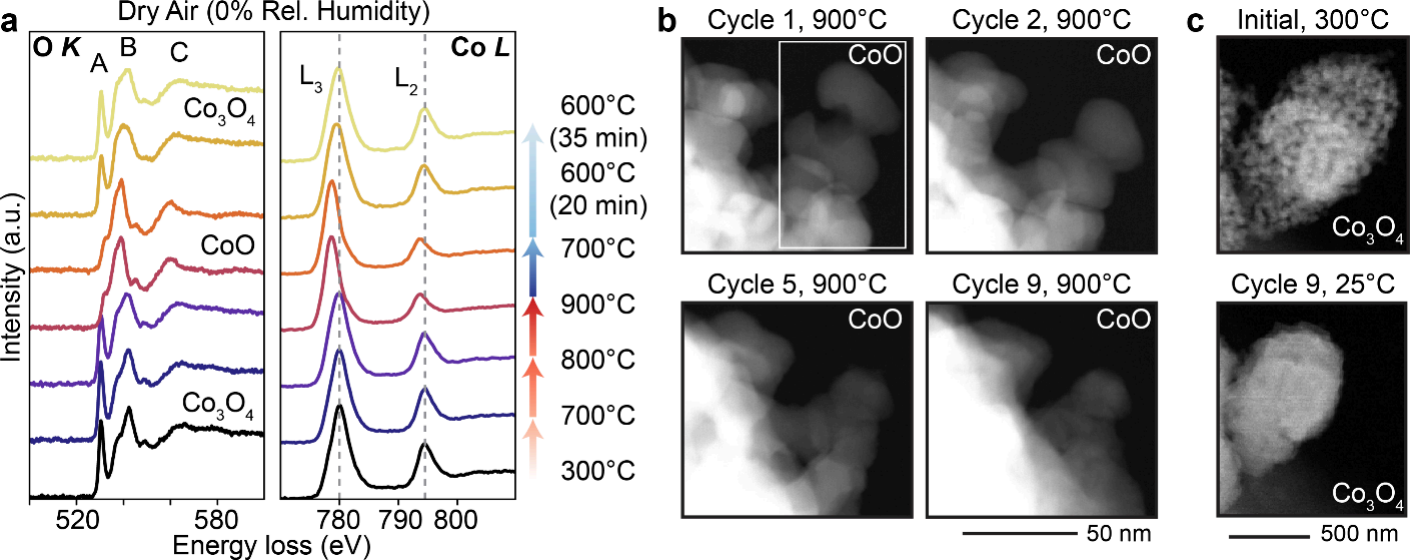
Thermochemical cycling of cobalt oxide nanoparticles under dry air (0.5 mbar and 0% relative humidity). (a) Summed EEL spectra (from spectral images of the region outlined in (b)) at multiple temperature points in the first cycle, showing reduction at 900 °C and reoxidation starting at 600 °C. (b) ADF-STEM images captured in the reduced state during the first nine thermal cycles and (c) ADF-STEM images of the full cluster in the oxidized state before and after nine cycles. Significant densification into a single particle is observed within these nine cycles.

In summary, this work has demonstrated the powerful utility of environmental transmission electron microscopy for evaluating the thermal cycling behavior and degradation mechanisms of nanoscale thermochemical materials under realistic operating conditions. Using cobalt oxide as a representative metal oxide TCM, correlative, time-resolved EELS and STEM imaging revealed considerable degradation in the conversion kinetics after a single thermal cycle, resulting from particle sintering and nanostructural densification. In comparison, some published bulk thermal measurements on cobalt oxide microparticles have demonstrated reasonable stability over tens of cycles.^7,11^ The potential for faster conversion kinetics in nanoscale materials therefore competes with their propensity for sintering-driven densification. The change in kinetics and nanostructure were both most apparent during the reoxidation, possibly due to the volumetric expansion of the nanoparticles that occurs during this transformation. Analogous experiments in dry air revealed that the presence of water vapor affects the oxidation temperature but has a limited effect on the degree of sintering long-term. Going forward, these results point to synthetic and/or nanostructural design as the most impactful factors for achieving more durable nanoscale TCM systems for long-duration thermal energy storage. More broadly, the integration of operando ETEM and rapid-acquisition EEL spectral imaging described herein could be implemented for studying the interplay between morphological transformations and redox kinetics in many other nanostructured metal oxide systems for applications not only in energy storage but also in catalysis and advanced electronic devices and sensors.

## Supplementary Material



## Notes

Experimental methods (including materials and sample preparation, electron microscopy, and data processing), lower magnification images, MLLS fitting details, atomic composition analysis, discussion of spatial distribution of redox conversion, and volumetric density quantification (PDF)

## References

[1] Miró L., Gasia J., Cabeza L. F. (2016). Thermal Energy Storage (TES) for
Industrial Waste Heat (IWH) Recovery: A Review. Appl. Energy.

[2] Pelay U., Luo L., Fan Y., Stitou D., Rood M. (2017). Thermal Energy Storage
Systems for Concentrated Solar Power Plants. Renew. Sust. Energy.

[3] Faizan M., Alkaabi A. K., Nie B., Afgan I. (2024). Thermal Energy
Storage
Integration with Nuclear Power: A Critical Review. J. Energy Storage.

[4] Pardo P., Deydier A., Anxionnaz-Minvielle Z., Rougé S., Cabassud M., Cognet P. (2014). A Review on High Temperature
Thermochemical
Heat Energy Storage. Renew. Sust. Energy.

[5] Carrillo A. J., González-Aguilar J., Romero M., Coronado J. M. (2019). Solar energy
on demand: a review on high temperature thermochemical heat storage
systems and materials. Chem. Rev..

[6] Neises M., Tescari S., de Oliveira L., Roeb M., Sattler C., Wong B. (2012). Solar-heated rotary
kiln for thermochemical energy storage. Sol.
Energy.

[7] Liu L., Zhou Z., Wang C., Xu J., Xia H., Chang G., Liu X., Xu M. (2021). Superior Thermochemical
Energy Storage Performance of the Co_3_O_4_/CoO
Redox Couple with a Cubic Micro-Nanostructure. J. Energy Storage.

[8] Liu L., Zhou Z., Cao X. E., Zhou Y., Peng D., Liu Y., Liu X., Xu M. (2023). Screening of optimal dopants on cobalt-based
ceramics for high-temperature thermochemical energy storage. Ceram. Int..

[9] Han X., Wang L., Ge Z., Lin X., Liu Y., Zhang S., Zuo Z., Chen H. (2023). Al-and Cr-Doped
Co_3_O_4_/CoO Redox Materials for Thermochemical
Energy
Storage in Concentrated Solar Power Plants. Sol. Energy Mater. Sol. Cells.

[10] Yilmaz D., Darwish E., Leion H. (2020). Investigation of the
combined Mn-Si
oxide system for thermochemical energy storage applications. J. Energy Storage.

[11] Agrafiotis C., Roeb M., Schmücker M., Sattler C. (2014). Exploitation of Thermochemical
Cycles Based on Solid Oxide Redox Systems for Thermochemical Storage
of Solar Heat. Part 1: Testing of Cobalt Oxide-Based Powders. Sol. Energy.

[12] Wu R., Huang H., Deng L., Kubota M., Kobayashi N. (2023). Influence
of CuO Doping on Cobalt Oxide for Thermochemical Energy Storage. Sol. Energy Mater. Sol. Cells.

[13] Wang L., Liu B., Ran S., Huang H., Wang X., Liang B., Chen D., Shen G. (2012). Nanorod-assembled Co_3_O_4_ Hexapods with Enhanced
Electrochemical Performance for Lithium-Ion
Batteries. J. Mater. Chem..

[14] Rai A. K., Gim J., Anh L. T., Kim J. (2013). Partially Reduced Co_3_O_4_/Graphene Nanocomposite
as an Anode Material for Secondary
Lithium Ion Battery. Electrochim. Acta.

[15] Carrillo A. J., Serrano D. P., Pizarro P., Coronado J. M. (2014). Thermochemical Heat
Storage Based on the Mn_2_O_3_/Mn_3_O_4_ Redox Couple: Influence of the Initial Particle Size on the
Morphological Evolution and Cyclability. J.
Mater. Chem. A.

[16] Zhao Y., Feltes T. E., Regalbuto J. R., Meyer R. J., Klie R. F. (2010). In Situ
Electron Energy Loss spectroscopy Study of Metallic Co and Co Oxides. J. Appl. Phys..

[17] Xin H. L., Pach E. A., Diaz R. E., Stach E. A., Salmeron M., Zheng H. (2012). Revealing Correlation of Valence State with Nanoporous Structure
in Cobalt Catalyst Nanoparticles by In Situ Environmental TEM. ACS Nano.

[18] Ward M. R., Boyes E. D., Gai P. L. (2013). In Situ Aberration-Corrected Environmental
TEM: Reduction of Model Co_3_O_4_ in H_2_ at the Atomic Level. ChemCatChem..

[19] Chen X., Van Gog H., Van Huis M. A. (2021). Transformation
of Co_3_O_4_ Nanoparticles to CoO Monitored by In
Situ TEM and Predicted
Ferromagnetism at the Co_3_O_4_/CoO Interface from
First Principles. J. Mater. Chem. C.

[20] Ma C., Yun Y., Zhang T., Suo H., Yan L., Shen X., Li Y., Yang Y. (2021). Insight into
the Structural Evolution of the Cobalt
Oxides Nanoparticles upon Reduction Process: An In Situ Transmission
Electron Microscopy Study. ChemCatChem..

[21] Wei Y., Zhang Z., Mei C., Tan J., Wang Z., Li J., Gan L. (2023). Facet Dependence and
Vacancy-Controlled Reversibility
of the Spinel-to-Rocksalt Phase Transformation at Co_3_O_4_ Surfaces. Chem. Mater..

[22] Ha D.-H., Moreau L. M., Honrao S., Hennig R. G., Robinson R. D. (2013). The Oxidation
of Cobalt Nanoparticles into Kirkendall-Hollowed CoO and Co_3_O_4_: The Diffusion Mechanisms and Atomic Structural Transformations. J. Phys. Chem. C.

[23] Zhang D., Jin C., Li Z., Zhang Z., Li J. (2017). Oxidation Behavior
of Cobalt Nanoparticles Studied by In Situ Environmental Transmission
Electron Microscopy. Sci. Bull..

[24] Frati F., Hunault M. O., De Groot F. M. (2020). Oxygen
K-edge X-ray Absorption Spectra. Chem. Rev..

[25] Carrillo A. J., Serrano D. P., Pizarro P., Coronado J. M. (2015). Thermochemical Heat
Storage at High Temperatures using Mn_2_O_3_/Mn_3_O_4_ System: Narrowing the Redox Hysteresis by Metal
Co-doping. Energy Procedia.

[26] Wokon M., Block T., Nicolai S., Linder M., Schmücker M. (2017). Thermodynamic
and Kinetic Investigation of a Technical Grade Manganese-Iron Binary
Oxide for Thermochemical Energy Storage. Sol.
Energy.

[27] Block T., Knoblauch N., Schmücker M. (2014). The Cobalt-Oxide/Iron-Oxide Binary
System for Use as High Temperature Thermochemical Energy Storage Material. Thermochim. Acta.

[28] Carrillo A. J., Moya J., Bayon A., Jana P., de la Pena
O’Shea V. A., Romero M., Gonzalez-Aguilar J., Serrano D. P., Pizarro P., Coronado J. M. (2014). Thermochemical Energy
Storage at High Temperature via Redox Cycles of Mn and Co Oxides:
Pure Oxides Versus Mixed Ones. Sol. Energy Mater.
Sol. Cells.

[29] Żyła M., Smoła G., Knapik A., Rysz J., Sitarz M., Grzesik Z. (2016). The Formation of the Co_3_O_4_ Cobalt
Oxide within CoO Substrate. Corros. Sci..

[30] Ch’ng H., Pan J. (2007). Sintering of particles
of different sizes. Acta Mater..

[31] Agrafiotis C., Tescari S., Roeb M., Schmücker M., Sattler C. (2015). Exploitation of thermochemical cycles
based on solid
oxide redox systems for thermochemical storage of solar heat. Part
3: Cobalt oxide monolithic porous structures as integrated thermochemical
reactors/heat exchangers. Sol. Energy.

[32] Agrafiotis C., Tescari S., Roeb M., Schmücker M., Sattler C. (2015). Exploitation of thermochemical cycles
based on solid
oxide redox systems for thermochemical storage of solar heat. Part
2: Redox oxide-coated porous ceramic structures as integrated thermochemical
reactors/heat exchangers. Sol. Energy.

[33] Karagiannakis G., Pagkoura C., Halevas E., Baltzopoulou P., Konstandopoulos A. G. (2016). Cobalt/cobaltous oxide based honeycombs
for thermochemical
heat storage in future concentrated solar power installations: Multi-cyclic
assessment and semi-quantitative heat effects estimations. Sol. Energy.

[34] Heli H., Pishahang J. (2014). Cobalt oxide nanoparticles anchored
to multiwalled
carbon nanotubes: synthesis and application for enhanced electrocatalytic
reaction and highly sensitive nonenzymatic detection of hydrogen peroxide. Electrochim. Acta.

[35] Kumar R., Singh R. K., Dubey P. K., Singh D. P., Yadav R. M. (2015). Self-assembled
hierarchical formation of conjugated 3D cobalt oxide nanobead-CNT-graphene
nanostructure using microwaves for high-performance supercapacitor
electrode. ACS Appl. Mater. Interfaces.

[36] Li C., Zhang C., Guo X. (2023). Sintering
Mechanism of CaO During
Carbonation Reaction in the Presence of Water Vapor. Proc. Combust. Inst..

[37] Navrotsky A., Ma C., Lilova K., Birkner N. (2010). Nanophase
Transition Metal Oxides
Show Large Thermodynamically Driven Shifts in Oxidation-Reduction
Equilibria. Science.

[38] Bielsa D., Zaki A., Arias P. L., Faik A. (2020). Improving the redox
performance of Mn_2_O_3_/Mn_3_O_4_ pair by Si doping to be used as thermochemical energy storage for
concentrated solar power plants. Sol. Energy.

[39] Wang L., Wang L., Meng X., Xiao F.-S. (2019). New strategies for
the preparation of sinter-resistant metal-nanoparticle-based catalysts. Adv. Mater..

