# Supplementary material for: Revealing Progressive Degradation of Cobalt Oxide Nanoparticles During Thermochemical Redox Cycling via Operando STEM-EELS
Source: Nano Lett. 2025 Dec 17;25(52):18075–82. doi: 10.1021/acs.nanolett.5c05081 (PMC12766727; doi:10.1021/acs.nanolett.5c05081)
Supplement: Supplementary file 1 [file nl5c05081_si_001.pdf]

Supporting Information for

Revealing Progressive Degradation of Cobalt Oxide Nanoparticles During Thermochemical Redox Cycling via Operando STEM-EELS

Madeline Van Winkle,[†] Stephen D. House,[‡] Yuxiang Peng,[¶] Yu-chen Karen Chen-Wiegart,[¶,§] Katherine Jungjohann,[†] and John S. Mangum[*,†]

†*Materials Science Center, National Laboratory of the Rockies (formerly National Renewable Energy Laboratory), Golden, Colorado 80401, United States*

‡*Center for Integrated Nanotechnologies, Sandia National Laboratories, Albuquerque, New Mexico 87123, United States*

¶*Department of Materials Science and Chemical Engineering, Stony Brook University, Stony Brook, New York 11790, United States*

§*National Synchrotron Light Source II (NSLS-II), Brookhaven National Laboratory, Upton, New York 11973, United States*

E-mail: john.mangum@nrel.gov

Contents

1 Experimental methods **3**

1.1 Materials and sample preparation 3

1.2 Electron microscopy 3

1.3 Data processing 4

2 Full cluster images **5**

3 Multiple linear least squares fitting **6**

4 Atomic composition analysis **9**

5 Spatial distribution of redox conversion **11**

6 Volumetric density quantification **13**

1 Experimental methods

1.1 Materials and sample preparation

A dilute solution of Co_3O_4 nanoparticle powder (<50 nm diameter, Sigma-Aldrich, 99.5% purity) briefly sonicated (~1 min) in isopropanol was drop cast onto Protochips Fusion Select Heating E-chips with a silicon nitride support layer (~40 nm reported thickness). The chips were left to dry under ambient conditions, leaving dispersed nanoparticle clusters for imaging and spectroscopic analysis. Directly prior to the thermochemical cycling experiments, samples were heated in the microscope at 300°C for 30 min under the desired gas conditions to remove organic surface contaminants.

1.2 Electron microscopy

All microscopy was performed at the Center for Integrated Nanotechnologies user facility at Sandia National Laboratories (Albuquerque, NM) using a Thermo Fisher Scientific Titan Environmental TEM (ETEM) equipped with a custom-built gas delivery system. Most experiments (Figures 1–4 in the main text) were conducted under 0.5 mbar of Ultra Zero (UZ) grade air (i.e. very dry and clean air) purchased from Matheson Tri Gas, that was flowed through the headspace of a DI water-containing bubbler to increase the relative humidity to 38%. The relative humidity was calculated according to the following expression:

$$RH = \frac{P_{100}}{P} * 100\% \tag{1}$$

where P_{100} is the natural vapor pressure in the headspace with no added carrier gas (~20 Torr for water) and P is the total pressure in the headspace with carrier gas added (set to 53 Torr in this case). For the experiments conducted in dry conditions (Figure 5), pure UZ air was used (0% relative humidity).

Electron energy loss spectroscopy (EELS) was acquired using a Gatan K3-IS direct electron detector with a Gatan BioContinuum HD-HR imaging filter operated in DualEELS mode. The ETEM was operated at 300 kV with a 9.7 mrad convergence semi-angle and a 40.2 mrad collection semi-angle. For all data acquisition, the approximate beam current was 200–300 pA. This beam current provided sufficient spectral signal to achieve a temporal resolution that was useful for monitoring kinetics in this system using in-situ EELS. Lower beam currents could be feasibly used at the expense of temporal resolution. To identify the reduction and oxidation temperatures (Figure 1d, 5a), EEL spectral images of the regions of interest were collected with an exposure time of 50 or 100 ms with a 1 or 2 nm step size and a 8 x 8 pixel subscan with a total acquisition time of 8–9 min per spectral image. For the time-resolved experiments shown in Figures 2 and 3, EEL spectral images were recorded in in-situ mode in the Gatan Digital Micrograph software with an exposure time of 1 ms over 32 x 54 pixels with a 4 nm step size and 8 x 8 pixel subscan, yielding a total acquisition time of 2.4 s per spectral image. All STEM images shown were acquired using an ADF detector between or during collection of EELS datasets.

1.3 Data processing

Standard EEL spectral images were processed using the HyperSpy Python package.[1] The spectra shown in Figure 1d, 5a were generated by summing the spectra from all pixels in each spectral image, cropping the signal around the O K- and Co L-edges and subtracting the background using a power law fit.

In-situ EELS data sets were partially processed using Gatan Digital Micrograph software, including binning spectral images over time and/or space, performing multiple linear least squares (MLLS) fitting (Supplementary Figure 3), measuring relative thickness (Supplementary Figures 6b,e and 7b), and calculating areal Co density (Supplementary Figure 8). Further analyses and data visualization were implemented using custom Python scripts with HyperSpy, matplotlib, and seaborn packages.

2 Full cluster images

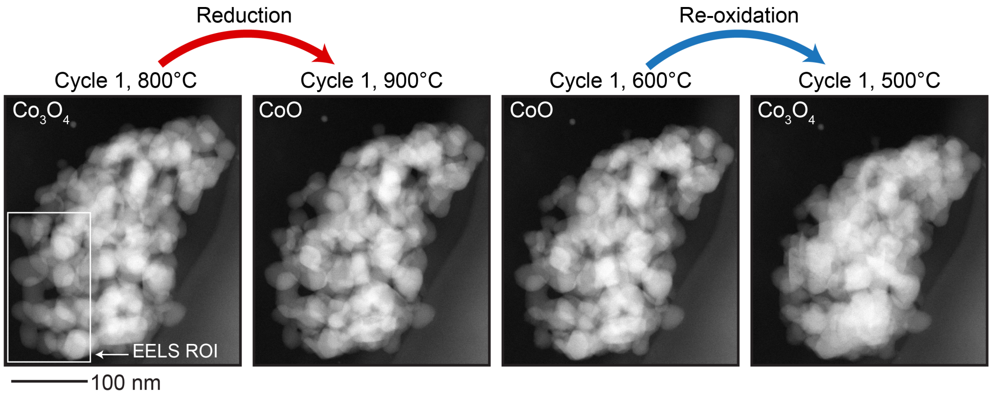

Supplementary Figure 1. Lower magnification ADF-STEM images of the cobalt oxide nanoparticle cluster at various temperatures during the first thermochemical cycle in humid air. Morphological changes are consistent in both the region sampled during EELS measurements (higher total beam dose) and the surrounding region only exposed for the single-pass ADF-STEM images shown (lower total beam dose), and there are no significant signs of beam damage in either region. This suggests that, under the conditions used in this work, beam exposure did not have a noticeable effect on the transition temperatures and extent of sintering observed. For reference, the dwell time was 50 ms/pixel for standard EELS spectral imaging, 1 ms/pixel for in-situ EELS spectral imaging ($\sim$250 ms total/pixel for all frames in a 10 min mapping period), and 16 μs/pixel for ADF-STEM.

3 Multiple linear least squares fitting

The fraction of CoO in the nanoparticle cluster was quantified using a multiple linear least squares fitting in the Gatan Digital Micrograph software. With this method, the relative contributions of Co_3O_4 and CoO to a given spectrum are determined by fitting to a linear combination of Co_3O_4 and CoO reference spectra. In this work, internal references (Supplementary Figure 2) were used to ensure acquisition parameters, instrument alignment, and atmospheric conditions were as similar to that of the data being fitted as possible. The fitting process itself consisted of first subtracting the pre-edge background near the Co L-edge with a power law fit, then fitting the two references to the remaining Co L-edge signal with a fitting window of 650–815 eV (Supplementary Figure 3). The fraction of CoO was quantified as follows:

$$\text{Fraction of CoO} = \frac{I_{CoO}}{I_{Co_3O_4} + I_{CoO}} \tag{2}$$

where I_{CoO} and $I_{Co_3O_4}$ are the signal integrals for CoO and Co_3O_4 from the resulting model. While the Co:O ratio can also be quantified directly from the relative computed cross sections of the Co L-edge and O K-edge signals, we found that the background O signal from the gaseous atmosphere reduced the accuracy of the O quantification. The MLLS fitting of the Co signal was therefore a more reliable approach for quantifying phase fraction.

For the data plotted in Figure 3 in the main text, the in-situ dataset was first binned by a factor of 5 over the time domain. All pixels in each binned spectral image were then summed, and the summed spectra were fit via MLLS (Method 1). By summing all pixels in each spectral image prior to fitting, the signal-to-noise ratio in the resulting spectra were sufficient for fitting without sacrificing much temporal resolution. However, given that there is variation in thickness throughout the cluster, the summed spectra may be skewed to primarily reflect contributions from thicker regions. With this in mind, we investigated two other quantification methods that could account for these variations more thoroughly. For Method 2, the in-situ data set was first binned by a factor of 15 over time and by a

factor of 4 in space in order to produce single-pixel spectra with sufficient signal-to-noise for fitting. The individual pixels in each binned spectral image (excluding background pixels) were then fit via MLLS and the resulting CoO fractions were averaged to give the overall average CoO fraction in the cluster. With Method 3, the data were processed and fit using the same procedure as Method 2, but the overall CoO fraction was calculated using a weighted average, where the weight of each pixel was the relative thickness computed from the corresponding low-loss signal. The results of these three quantification methods are plotted in Supplementary Figure 4 for the first and second reduction and oxidation cycles. Overall, we find that directly accounting for thickness variation throughout the cluster can shift the calculated CoO fraction by a small amount, typically <5%, but the kinetic trends do not change. Based on this analysis, we chose to report Method 1, which has the highest temporal resolution, in the main text.

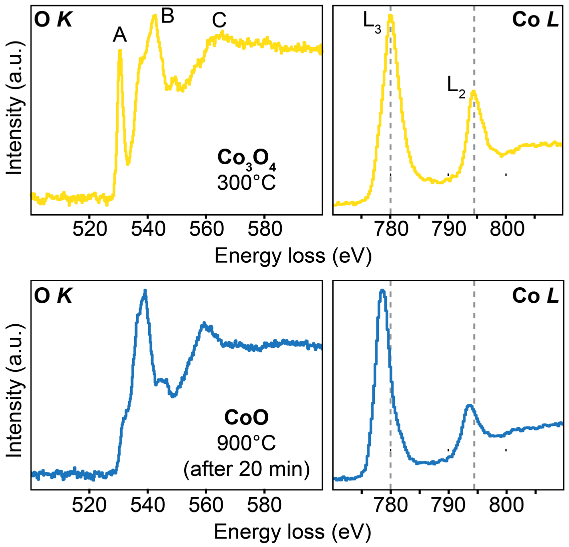

Supplementary Figure 2. Internal Co_3O_4 (top) and CoO (bottom) EELS references. The Co L-edges were used for MLLS fitting.

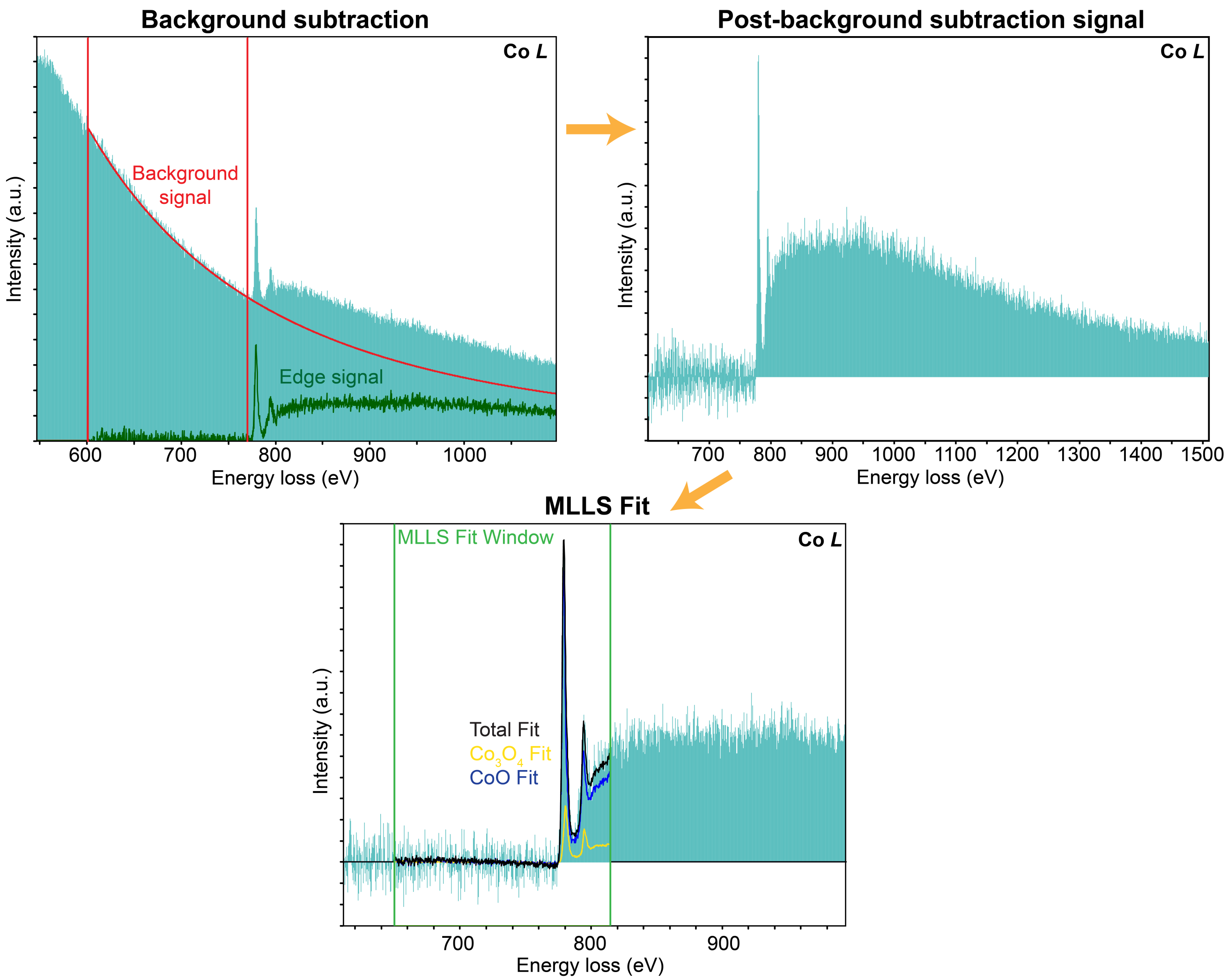

Supplementary Figure 3. MLLS fitting process using the Co $L_{3,2}$-edge and internal Co_3O_4 and CoO references.

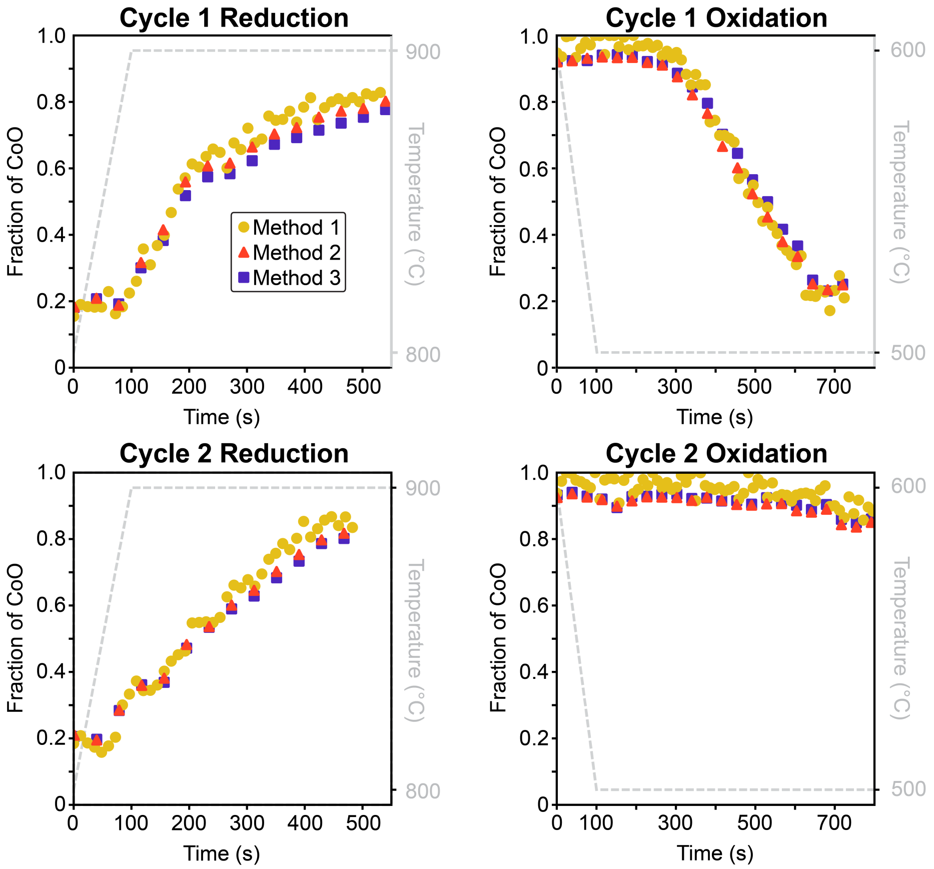

Supplementary Figure 4. Comparison of three quantification approaches for calculating fraction of CoO in the cluster. Method 1 sums all spectra in each spectral image prior to MLLS fitting, while Methods 2 and 3 individually fit each pixel in the spectral images and then either average the results (Method 2) or calculate a weighted average with the local relative thickness as the weight (Method 3). The three methods yield the same kinetic trends, with small deviations typically less than 5%.

4 Atomic composition analysis

The relative atomic percentages of Co and O were calculated for each in-situ EELS dataset using theoretical cross-sections in Gatan Digital Micrograph (described in more detail in Section 6 of the Supporting Information). Supplementary Figure 5 shows the percent change in average Co and O content within the cluster during the first two cycles. From this analysis, we observe a decrease in O content in the cluster during reduction and an increase during reoxidation. Minimal change in O content is observed during the second reoxidation due to

particle sintering, which impeded reaction progress. The presence of oxygen in the gaseous atmosphere surrounding the sample does affect the quantitative accuracy of this measurement. However, the oxygen content in the area outside of the cluster varies randomly during cycling (Supplementary Figure 5c) whereas the oxygen content within the cluster shows clear trends (Supplementary Figure 5b). Therefore the observed trends are representative of oxygen uptake and release from the cobalt oxide itself.

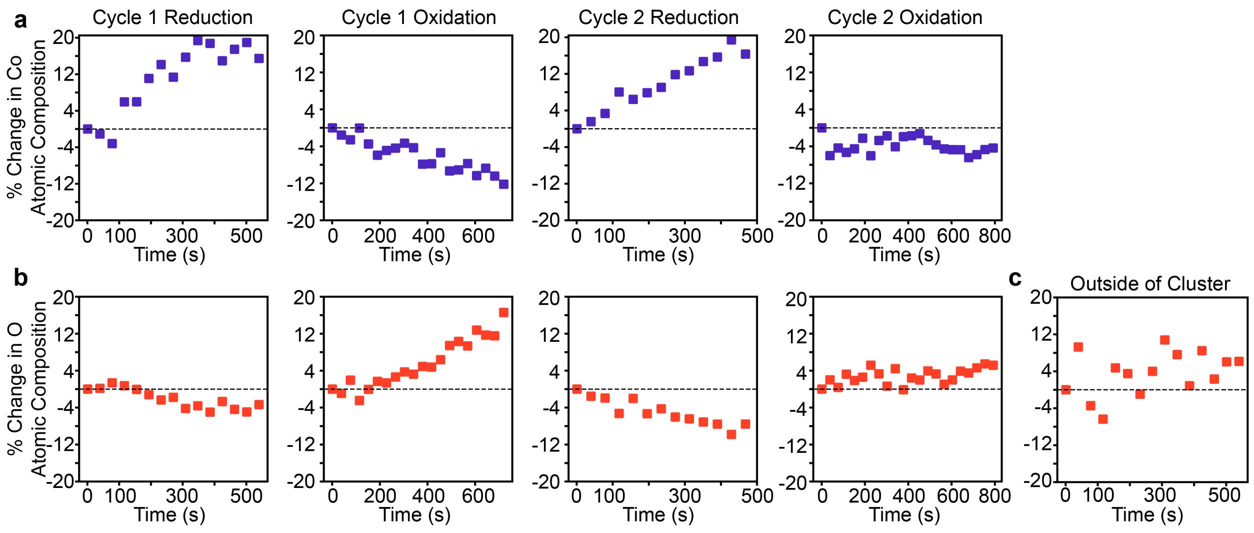

Supplementary Figure 5. Percent change in average (a) Co and (b) O atomic composition within the cluster during the first two thermochemical cycles, showing a decrease in O content during reduction and an increase during oxidation. (c) Percent change in average O atomic composition in the area surrounding the cluster during the first reduction cycle, showing random variation in the oxygen background signal.

5 Spatial distribution of redox conversion

During the first thermochemical cycle, reduction and reoxidation both nucleate in similar parts of the cluster, as shown in Figure 2 in the main text. We hypothesize that this is a statistical effect, where regions of the cluster with a higher density of overlapping nanoparticles convert first simply due to the presence of more more nanoparticles. Supplementary Figure 6 shows the ADF-STEM images, relative thickness maps calculated using the log-ratio method[2] with the low-loss EELS data, and Co_3O_4/CoO phase maps illustrating where reduction and reoxidation nucleate during the first cycle. Based on this data, thicker parts of the cluster (i.e. areas with more particles) do convert first, lending validity to our hypothesis. Notably, this effect is less pronounced in the second cycle (Supplementary Figure 7). As shown in Supplementary Figure 7c, reduction starts and spreads more uniformly throughout the cluster in the second cycle, likely because the sintering that occurred in the first cycle generated a more even distribution of exposed surface within the cluster.

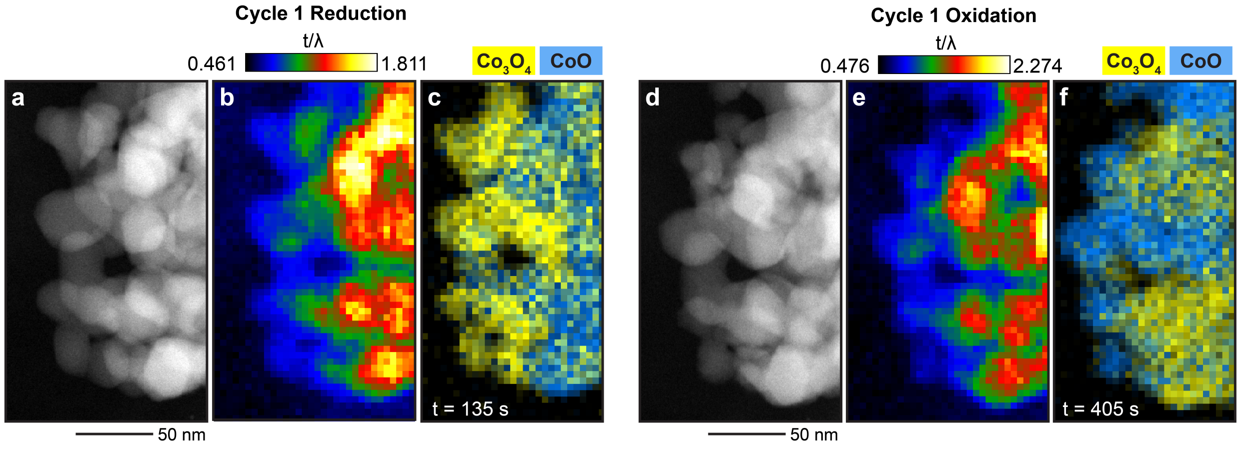

Supplementary Figure 6. (a,d) Initial ADF-STEM images, (b,e) relative thickness maps, and (c,f) Co_3O_4/CoO phase maps at specified time points during the first (a,b,c) reduction and (c,d,e) reoxidation, showing that both reduction an reoxidation nucleate in thicker parts of the cluster with more particles.

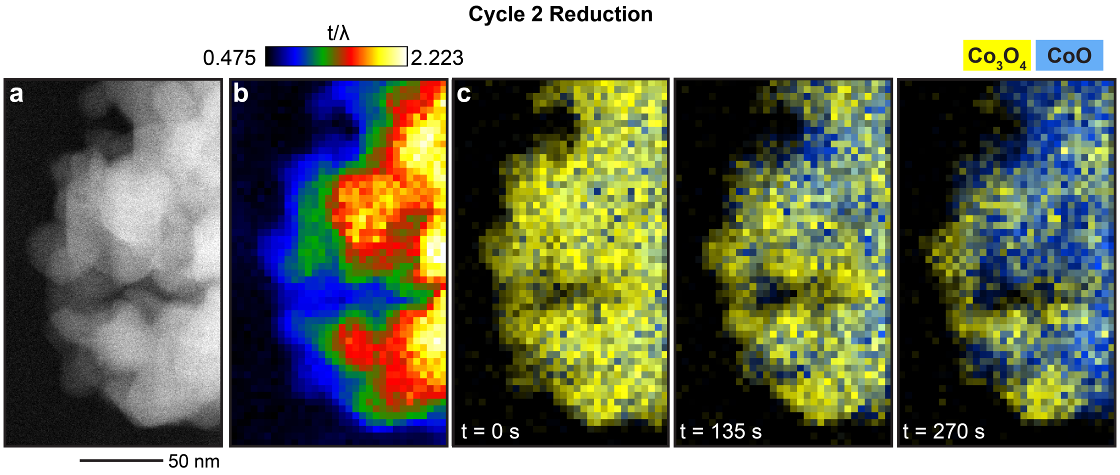

Supplementary Figure 7. (a) Initial ADF-STEM image, (b) relative thickness map, and (c) Co_3O_4/CoO phase maps at specified time points during the second reduction cycle, showing more uniformly dispersed reduction over time due to sintering in the first cycle.

6 Volumetric density quantification

To quantify the volumetric density of Co atoms in the cluster, we first calculated the areal density of Co atoms (the number of Co atoms per nm^2) using the EELS Quantification feature in the Gatan Digital Micrograph software. In brief, this feature models the EEL spectrum at each pixel in the acquired spectral images using a power law background and theoretical cross-section for the element edge of interest while also taking into account plural scattering effects.[3] An example fit is shown in Supplementary Figure 8. The areal density of Co atoms is automatically extracted based on the integrated edge signal in the model fit.

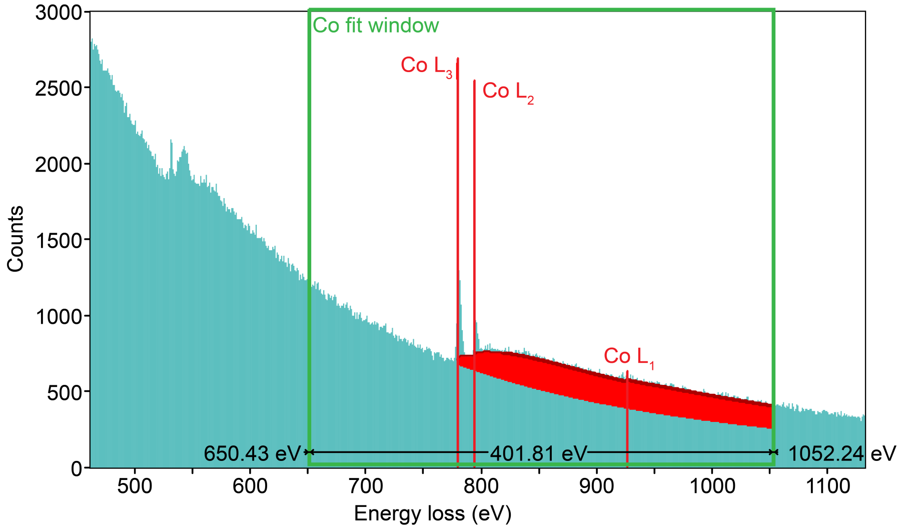

Supplementary Figure 8. Example model fit using a power law background and theoretical Hartree Slater cross-section of the Co L-edge. The integrated edge signal (red) relates to the number of Co atoms per nm^2.

The areal density (d_a) was then divided by the local thickness of the sample according to the following expression to yield the volumetric density (d_v, number of Co atoms per nm^3):

$$d_v = \frac{d_a}{(\lambda * \frac{t}{\lambda}) - t_{membrane}} \tag{3}$$

Here, t/λ is the relative total thickness calculated using the log-ratio method with the low-loss EELS data, reported in terms of the number of inelastic mean free paths (IMFP), λ.[2]

The absolute total thickness (t, in nm) is estimated by multiplying by λ for Co_3O_4, which is around 112 nm based on the equations provided by Egerton and Cheng.[2] Lastly, we subtract the approximate thickness of the silicon nitride membrane ($t_{membrane}$) to isolate the thickness of the Co-containing material. Some error is expected in the volumetric density calculation due to the approximations made for the total thickness and membrane thickness; however, we expect the general trends shown to be negligibly affected. For example, the error in the IMFP calculated from the log-ratio method can be up to $\sim$10%, but would be the same throughout all the calculations here and thus not affect the relative comparisons.

We note that the membrane surrounding the cluster was masked off prior to the volumetric density calculations based on a cutoff value of 700 at./nm^2 for the areal Co density. Visually, this cutoff value sufficiently isolated the membrane without masking material in the cluster. The final regions included in the volumetric density calculation, which include intra-cluster voids/porosity, are shown in the maps in Figure 4d of the main text.

References

(1) de la Peña, F. et al. Hyperspy. Ver 2.3.0, 2025; https://doi.org/10.5281/zenodo.14956374.

(2) Egerton, R.; Cheng, S. Measurement of Local Thickness by Electron Energy-Loss Spectroscopy. *Ultramicroscopy* **1987**, *21*, 231–244.

(3) Gatan. Quantify Extracted Signal. https://eels.info/how/quantification/quantify-extracted-signal#How%20To.
